# Responsiveness of human bronchial fibroblasts and epithelial cells from asthmatic and non-asthmatic donors to the transforming growth factor-β_1_ in epithelial-mesenchymal trophic unit model

**DOI:** 10.1186/s12860-021-00356-8

**Published:** 2021-03-12

**Authors:** Milena Paw, Dawid Wnuk, Bogdan Jakieła, Grażyna Bochenek, Krzysztof Sładek, Zbigniew Madeja, Marta Michalik

**Affiliations:** 1 Department of Cell Biology, Faculty of Biochemistry, Biophysics and Biotechnology, Jagiellonian University, Gronostajowa 7, 30-378 Kraków, Poland; 2 Division of Molecular Biology and Clinical Genetics, Faculty of Medicine, Jagiellonian University Medical College, Kraków, Poland; 3 Department of Internal Medicine, Faculty of Medicine, Jagiellonian University Medical College, Kraków, Poland

**Keywords:** Transforming growth factor-β_1_, Air-liquid interface, Human bronchial epithelium, Human bronchial (myo)fibroblasts, Asthma, Epithelial-mesenchymal trophic unit

## Abstract

**Background:**

The asthma-related airway wall remodeling is associated i.a. with a damage of bronchial epithelium and subepithelial fibrosis. Functional interactions between human bronchial epithelial cells and human bronchial fibroblasts are known as the epithelial-mesenchymal trophic unit (EMTU) and are necessary for a proper functioning of lung tissue. However, a high concentration of the transforming growth factor-β_1_ (TGF-β_1_) in the asthmatic bronchi drives the structural disintegrity of epithelium with the epithelial-to-mesenchymal transition (EMT) of the bronchial epithelial cells, and of subepithelial fibrosis with the fibroblast-to-myofibroblast transition (FMT) of the bronchial fibroblasts. Since previous reports indicate different intrinsic properties of the human bronchial epithelial cells and human bronchial fibroblasts which affect their EMT/FMT potential beetween cells derived from asthmatic and non-asthmatic patients, cultured separatelly in vitro, we were interested to see whether corresponding effects could be obtained in a co-culture of the bronchial epithelial cells and bronchial fibroblasts. In this study, we investigate the effects of the TGF-β_1_ on the EMT markers of the bronchial epithelial cells cultured in the air-liquid-interface and effectiveness of FMT in the bronchial fibroblast populations in the EMTU models.

**Results:**

Our results show that the asthmatic co-cultures are more sensitive to the TGF-β_1_ than the non-asthmatic ones, which is associated with a higher potential of the asthmatic bronchial cells for a profibrotic response, analogously to be observed in '2D' cultures. They also indicate a noticeable impact of human bronchial epithelial cells on the TGF-β_1_-induced FMT, stronger in the asthmatic bronchial fibroblast populations in comparison to the non-asthmatic ones. Moreover, our results suggest the protective effects of fibroblasts on the structure of the TGF-β_1_–exposed mucociliary differentiated bronchial epithelial cells and their EMT potential.

**Conclusions:**

Our data are the first to demonstrate a protective effect of the human bronchial fibroblasts on the properties of the human bronchial epithelial cells, which suggests that intrinsic properties of not only epithelium but also subepithelial fibroblasts affect a proper condition and function of the EMTU in both normal and asthmatic individuals.

**Supplementary Information:**

The online version contains supplementary material available at 10.1186/s12860-021-00356-8.

## Background

Maintaining tissue homeostasis with normal physiology of human airways depends on the proper cooperation between different types of cells forming the respiratory tracts (epithelial cells, fibroblasts, smooth muscle cells, endothelial and immune cells). However, according to the theory by Plopper and Evans [1], proximity of bronchial epithelium and subepithelial fibroblasts largely coordinates the control of the airway microenvironment during lung development as well as in the progression of pathophysiological processes involving a tissue damage and repair or regulation of inflammation [2]. Immediate anatomical and functional interactions between airway epithelial cells and fibroblasts are known as the epithelial-mesenchymal trophic unit (EMTU) [1]). Alterations in its functionality observed in many respiratory disorders such as idiopathic pulmonary fibrosis (IPF), a chronic obstructive pulmonary disease (COPD) or asthma lead to multiplications of signals to uncontrolled airway remodeling which causes irreversible changes and permanent impairment of the respiratory function of these patients [2–6].

Asthma is defined as a chronic heterogeneous disease affecting around 235 million people and running usually with airway inflammation [7]. In response to relatively high chronic local concentrations of proinflammatory and/or profibrotic cytokines including mainly the transforming growth factor-β (TGF-β), the airway structural cells undergo functional and phenotypic changes [8]. The TGF-β_1_-activated bronchial epithelium is disintegrated by down-regulation of tight junctions and undergoes the epithelial-mesenchymal transition (EMT) in which the epithelial markers (e.g. E-cadherin; *CDH1*) are replaced by the mesenchymal ones (N-cadherin; *CDH2*) [2, 9, 10]. The abnormally functioning epithelium observed in asthma is also the source of secreted pro-inflammatory/profibrotic cytokines affecting deeper parts of airways [11]. The activated mesenchymal cells establish secretory feedback mechanisms and affect the airway epithelium. Based on these observations Holgate created a concept that the impairment of the barrier function of the airway epithelium plays a central role in the progression of the airway remodeling and asthma symptoms [12]. Comparative studies on the properties of the pseudostratified bronchial epithelium isolated from asthmatic (AS) and non-asthmatic (NA) individuals were previously reported [13, 14]. However, the epithelium derived from asthmatic donors is characterized by an enhanced expression of inflammatory- and remodeling-related genes in comparison to the ones obtained from non-asthmatic donors [13, 15]. These intrinsic properties of the asthmatic airway epithelial cells affect the profibrogenic potential of the airway fibroblasts [16–19].

Longitudinal studies reveal that the subepithelial layer containing airway fibroblasts is also effectively activated by profibrotic cytokines, including the TGF-β_1_, to phenotypic shifts into highly contractile myofibroblasts (fibroblast-to-myofibroblast transition, FMT). These cells are characterized by overexpression of α-smooth muscle actin (α-SMA) incorporated into microfilament bundles, and hypersecretion of the extracellular matrix (ECM) proteins as tenascin C, collagens and fibronectin [20]. The excessive secretory activity of myofibroblasts leads to thickening of the subepithelial layer which effectively impairs the respiratory tract functions with its enhanced contractility [8].

Although the concept of Holgate et al. indicating that the bronchial airways remodeling is determined by the properties of the epithelium through interactions within the EMTU is commonly accepted, several reports describe remodeling that may take place without inflammation or may precede inflammation [21, 22]. It suggests that mesenchymal cells, especially bronchial fibroblasts, may play more important role in induction or progression of remodeling than it was previously thought. Our recent studies indicate that the bronchial fibroblasts isolated from asthmatic patients reveal different features than their non-asthmatic counterparts [23–28]. Therefore, our present study aims to assess the effect of the human bronchial fibroblasts (HBFs) and their intrinsic properties on the responsiveness of the human bronchial epithelial cells (HBECs) to the TGF-β_1_ using in vitro EMTU model. We investigated the integrity of the epithelial layer and properties of differentiated HBECs in the absence or presence of the TGF-β_1_ when cultured alone or co-cultured with the HBFs, compatible asthmatic (HBECs AS / HBFs AS, from the same patients) or non-asthmatic (NA/NA) or mixed (AS/NA or NA/AS) in vitro EMTU models, respectively. Since the ALI-cultured pseudostratified human airway epithelium contains several subpopulations of specialized epithelial cells [14, 29], we analyzed the gene expression markers of goblet (*MUC5AC*^+^), ciliated (*DNAH9*^+^) and basal cells (*P63*^+^), and the expression of the selected EMT-related genes such as mesenchymal markers *CDH2* (N-cadherin), *ACTA2*, and EMT-related transcription factors *SNAI1* and *SNAI2* in all experimental conditions.

Moreover, we also checked the effect of the HBECs from AS and NA individuals on the properties and the FMT potential of the HBFs cultured in the EMTU model in relation to our previous reports indicating different properties of the HBFs AS and NA in standard ‘2D’ culture. In the EMTUs we analyzed the expression of the TGF-β_1_-induced FMT-related genes such as *ACTA2* (encoding α-SMA, the main marker of myofibroblasts, *TAGLN* (encoding transgelin - a smooth muscle marker and unique actin-binding protein involved in asthma), *FN1* (fibronectin – ECM protein important in subepithelial fibrosis in asthma) and *TNC* (tenascin C – ECM protein, biomarker for asthma). We also analyzed the protective effects of the HBFs on the properties of the HBECs in the EMTU model.

## Results

### ALI medium does not change the profibrotic properties of the TGF-β_1_-treated HBFs derived from asthmatics

**Fig. 1:**
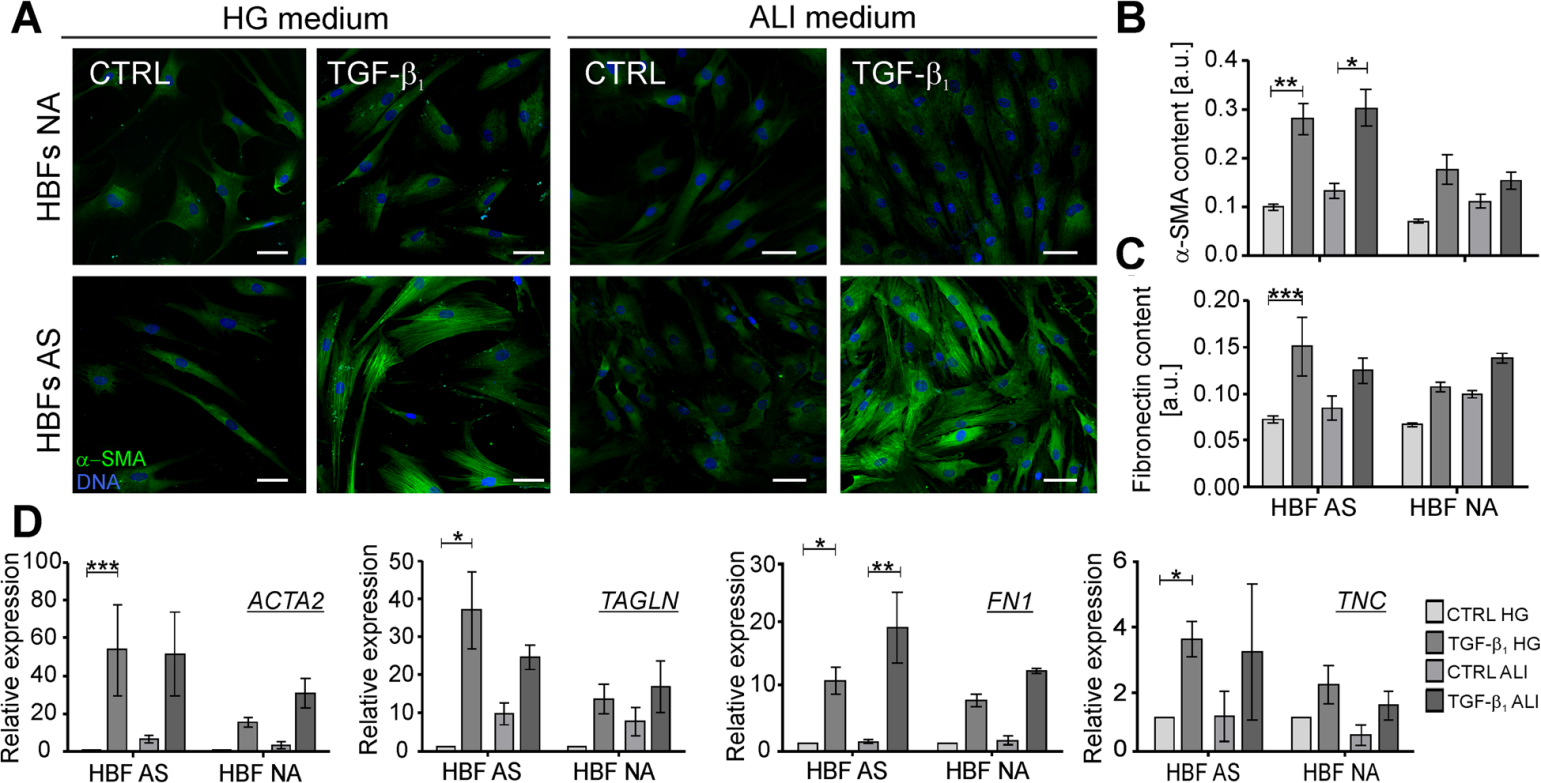
Profibrotic response of TGF-β_1_-stimulated HBFs remains unchanged in ALI medium. HBFs derived from asthmatic patients and non-asthmatic donors were cultured in HG medium or ALI medium in the absence (CTRL) or presence of TGF-β_1_ (5 ng/ml) for 4 days. Then, the FMT potential of HBFs was determined by **a** immunofluorescence (Bar = 50 μm), **b-c** *in-cell* ELISA test (each condition in triplicates) and **d** RT-qPCR. Expression of selected FMT-related genes: *ACTA2* (α-smooth muscle actin), *TAGLN* (transgelin), *FN1* (fibronectin) and *TNC* (tenascin C) was calculated using *GAPDH* as a reference gene and presented as 2^-ΔΔCt^ value. Data from all experiments represent the mean ± SEM carried out on HBFs AS (*n* = 4) and NA (*n* = 4). Statistical significance was tested using the Kruskall-Wallis with Dunn’s Multiple Comparison post hoc test (the comparison in one group). Statistical significance between tested groups (HBFs AS vs HBFs NA) was performed using the non-parametric Friedmann with Dunn’s Multiple Comparison post hoc test; * *p* < 0.05, ** *p* < 0.01, *** *p* < 0.001

Our longitudinal studies clearly show that populations of the HBF from AS and NA cultured in identical conditions in a serum-free medium display different features affecting their sensitivity to the external TGF-β_1_ and FMT potential [20, 28, 30]. In standard in vitro cultures the HBFs AS in response to the TGF-β_1_ develop the myofibroblastic phenotype characterized by a relatively high level of α-SMA incorporated into stress fibers more efficiently than the NA counterparts [23–25, 27, 28]. To clarify whether this phenomenon is pronounced in the EMTU model, firstly, we checked in this study if the medium dedicated for the air-liquid-interface (ALI) and EMTU cultures significantly affects the FMT potential of the TGF-β_1_-treated HBFs. For this purpose, we determined the myofibroblast phenotype in the HBF populations, derived from both investigated groups, by immunofluorescence and *in-cell* ELISA studies after 4 days of the HBFs culture in the serum-free medium with high glucose (HG) or ALI-dedicated medium (ALI), in the absence or presence of the TGF-β_1_. The obtained results indicated that the TGF-β_1_-treated HBFs from both NA and AS groups cultured in the ALI medium developed the myofibroblast populations in a degree similar to the cultures conducted in a standard HG medium (Fig. 1a). Moreover, effectiveness of myofibroblastic transitions observed in the HBF AS populations was significantly enhanced in contrast to the HBFs NA. The immunofluorescent studies were confirmed by the measurement of the α-SMA content incorporated into microfilaments (Fig. 1b) and the content of fibronectin (Fig. 1c) by *in-cell* ELISA tests. A distinct FMT potential of both types of the HBFs observed at the protein levels was confirmed also by RT-qPCR analyses of the FMT-related gene expression, as *ACTA2* (encoding α-SMA), *TAGLN* (transgelin), *FN1* (fibronectin) and *TNC* (tenascin C). The TGF-β_1_-inducible expression of the FMT-related genes in the HBF populations from AS or NA cultured in HG or ALI medium was comparable (Fig. 1d). These data clearly indicate that the medium dedicated for the ALI and EMTU cultures does not change the HBFs properties and their FMT potential in response to the TGF-β_1_ administration making it useful for the EMTU experimental model.

### EMTU cultures from asthmatics are more sensitive to the TGF-β_1_ than non-asthmatic ones

**Fig. 2:**
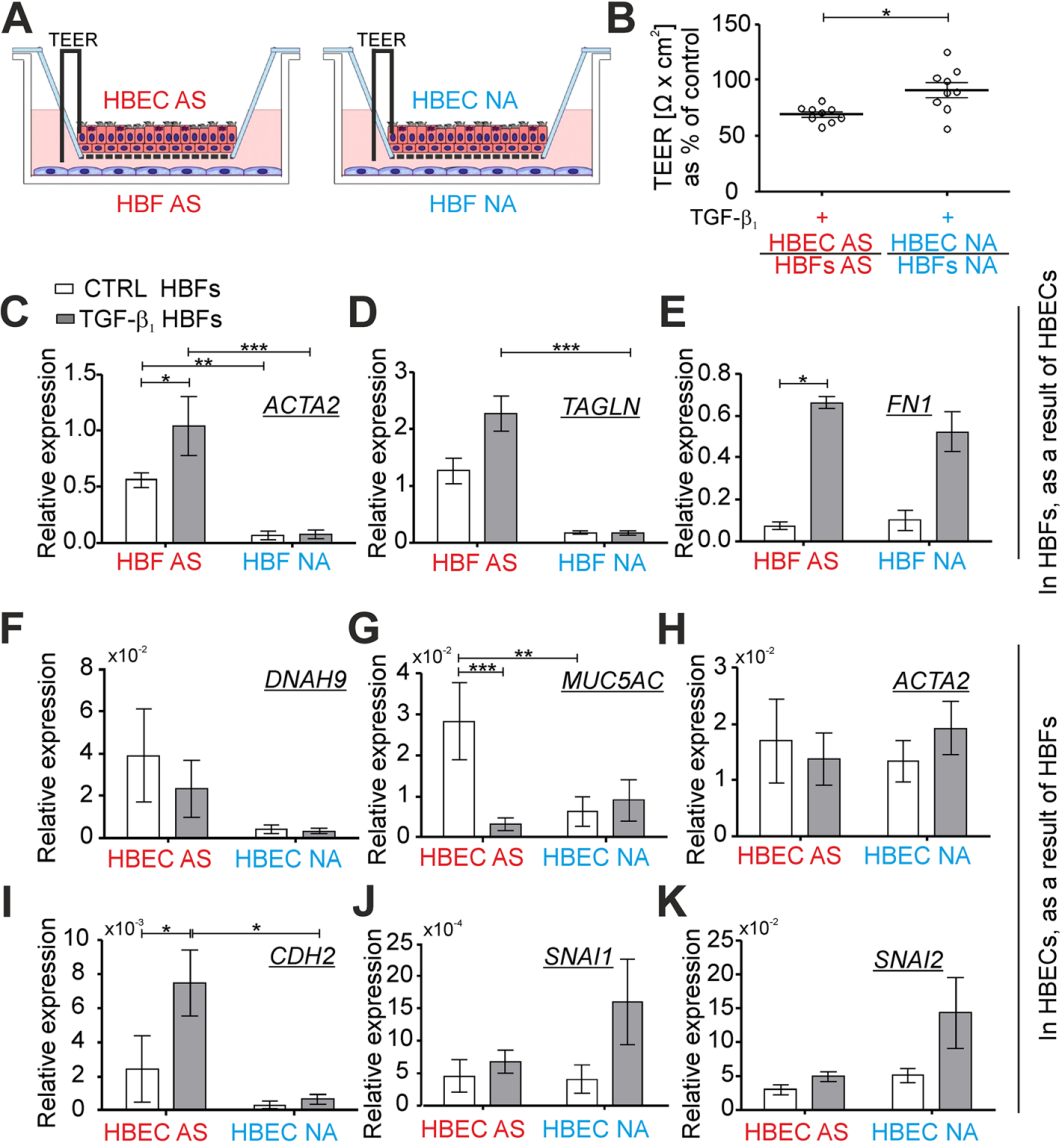
Asthmatic EMTUs are more sensitive on the TGF-β_1_ than non-asthmatic counterparts. **a** Mucociliary differentiated HBECs were cultured in the presence of HBF populations for 4 days in EMTU model in the absence (CTRL) or presence of TGF-β_1_ (5 ng/ml). TEER values were measured before EMTU establishment and after 4 days of cultures in triplicates for each experimental condition. **b** Results were presented as the change of TEER value after 4 days of EMTU culture in relation to TEER values before EMTU establishment (% of control, where the control group was compatible EMTU without the presence of TGF-β_1_) as an average ± SEM. Expression of selected **c-e** FMT- related genes: *ACTA2* (α-smooth muscle actin), *TAGLN* (transgelin), *FN1* (fibronectin); **f-g** markers of mucociliary differentiated HBECs: *DNAH9* (dynein axonemal heavy chain 9), *MUC5AC* (mucin 5 AC) and **h-k** EMT-related genes: *ACTA2*, *CDH2* (N-cadherin), *SNAI1* (Snail) and *SNAI2* (Slug) was measured by RT-qPCR and presented as 2^-ΔCt^ value in relation to *GAPDH* as a reference gene. Data from all experiments represent the mean ± SEM carried out on HBFs AS (*n* = 4) and NA (*n* = 4). Statistical significances were tested using the non-parametric Friedmann with Dunn’s Multiple Comparison post hoc test (comparison between AS and NA group) or Kruskall-Wallis tests (comparison within the group) with Dunn’s Multiple Comparison post hoc test; * *p* < 0.05, ** *p* < 0.01,*** *p* < 0.001

Different properties of the normal and asthmatic HBFs as well as the bronchial epithelium cultured independently in vitro were described previously [20, 31, 32]. In this study, we checked the properties and sensitivity of these cells from asthmatics and non-asthmatics growing in co-cultures AS/AS and NA/NA in in vitro EMTU model to the TGF-β_1_ (Fig. 2a). Firstly, we investigated the integrity of the mucociliary differentiated HBECs grown on Transwell inserts by the measurement of transepithelial electrical resistance (TEER) [33, 34] before EMTU establishment. The TEER value was measured again in triplicates in each insert containing differentiated HBECs cultured in the presence of HBFs (in the EMTU) for 4 days. Our results presented as a change in the TEER value in HBECs after EMTU establishment (after 4 days) in relation to the TEER values before EMTU establishment (control, in 0 day) show a significant decrease of the TEER values in both types of the EMTU (AS/AS and NA/NA) after the TGF-β_1_ treatment (Fig. S1). However, the AS co-cultures are more sensitive to the addition of exogenous TGF-β_1_ than the NA ones (Fig. 2b). Next, we analyzed the effectiveness of the FMT at the mRNA levels using RT-qPCR in HBF populations cultured in the EMTU model after administration of the TGF-β_1_. Our results revealed that the HBFs AS show the enhanced level of *ACTA2* (the main marker of FMT) in the non-treated AS/AS EMTU in comparison to the NA counterparts in co-cultures NA/NA (Fig. 2c). Moreover, the expression of *ACTA2* and *TAGLN* increased significantly following stimulation with the TGF-β_1_ only in the AS/AS EMTU co-culture (Fig. 2c, d). Analyses of the fibronectin expression revealed less spectacular differences between the HBFs from AS and NA groups, but the trend showing the increased FMT-related genes and FMT potential in asthmatics is maintained (Fig. 2e). Furthermore, we analyzed the response of the mucociliary differentiated ALI cultured HBECs to the addition of the exogenous TGF-β_1_. While the expressions of dynein axonemal heavy chain 9 gene (*DNAH9;* Fig. 2f) in the epithelial layer of the AS/AS EMTU co-cultures was similar to that of the NA/NA counterparts, a significantly higher expression of mucin 5 AC gene (*MUC5AC)* was observed in the AS epithelial layer in the control condition (Fig. 2g). The exogenous TGF-β_1_ caused a significant decrease of the *MUC5AC* expression only in the AS/AS EMTU (Fig. 2g) but had no substantial effect on the expression of *DNAH9* and *ACTA2* in the epithelial layer from both tested groups (Fig. 2f, h). Because the TGF-β_1_ is known as a potent activator of the EMT [10], we also analyzed the expression of *CDH2*, *SNAI1* and *SNAI2* in the HBECs ALI cultures in the EMTU model. TGF-β_1_-induced enhancement of the mesenchymal marker *CDH2* expression was significantly higher in the AS epithelium in the AS/AS EMTU than in the epithelial layer cultured in the NA/NA EMTU (Fig. 2i). The baseline expression of the EMT-related zinc finger transcription factors *SNAI1* and *SNAI2* was comparable in both tested models, but insignificant enhancement of these genes was observed in the NA/NA EMTU after exposition to the TGF-β_1_ (Fig. 2j-k). Our results indicate that the AS/AS EMTUs are more sensitive to the TGF-β_1_ than NA/NA ones. It is associated with a higher potential of asthmatic co-cultures for a profibrotic response, which is analogous to ‘2D’ standard in vitro cultures.

### Mucociliary differentiated HBECs efficiently diminish the TGF-β_1_–induced FMT potential in HBF populations

**Fig. 3:**
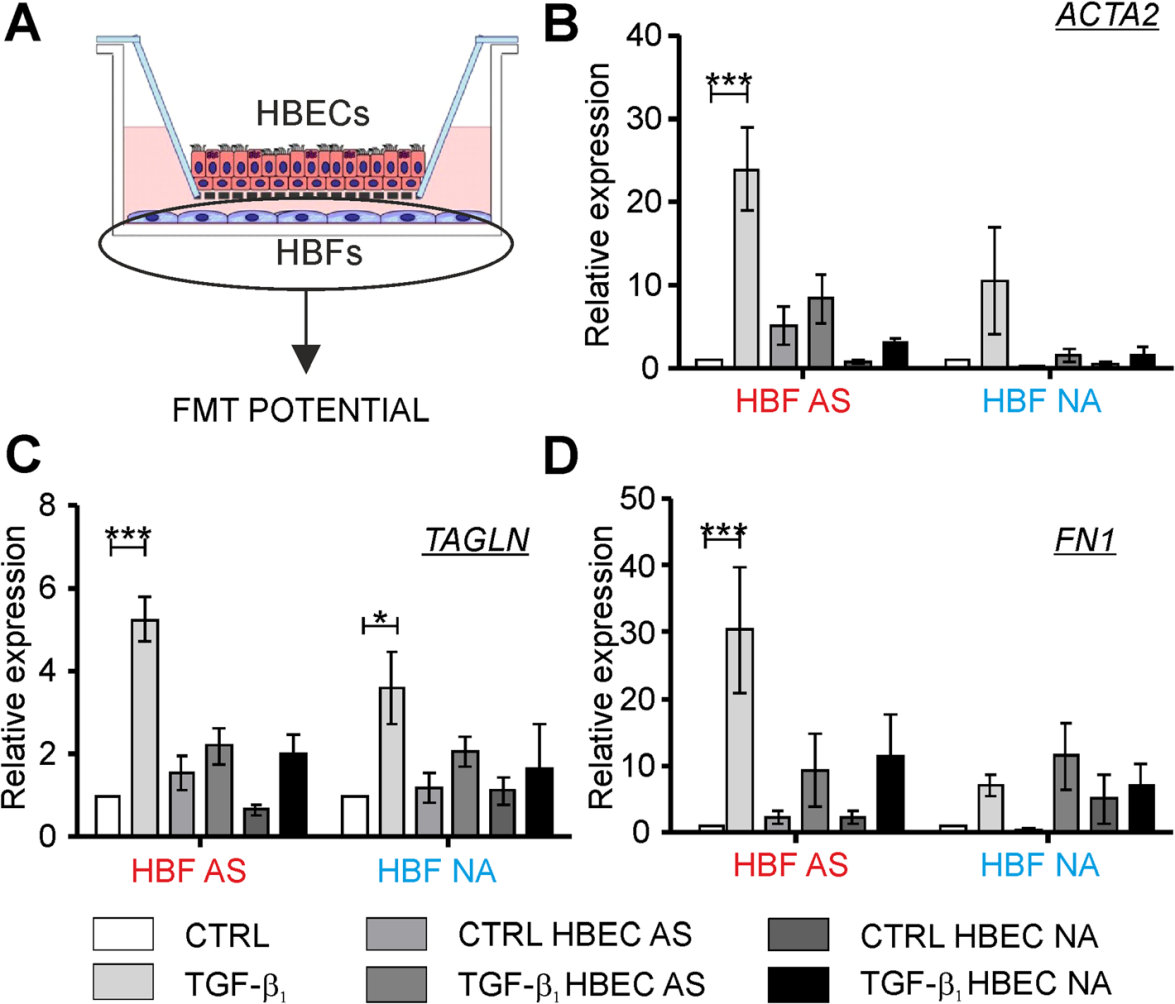
The profibrotic response of TGF-β_1_-treated HBFs is diminished by mucociliary differentiated HBECs in EMTU co-cultures. **a** HBFs derived from asthmatic patients (AS) and non-asthmatic donors (NA) were cultured alone (CTRL, TGF-β_1_) or in EMTU co-culture in the absence (CTRL HBEC) or presence of TGF-β_1_ (5 ng/ml; TGF-β_1_ HBEC) for 4 days. Then, the FMT potential of HBFs was determined by RT-qPCR using *GAPDH* as a reference gene. **b-d** *ACTA2*, *TAGLN* and *FN1* (FMT-related genes) expression levels in HBFs were presented as 2^-ΔΔCt^ value. Data from all experiments represent the mean ± SEM carried out on HBFs AS (*n* = 4) and NA (*n* = 4). Statistical significances were tested using the non-parametric Friedmann with Dunn’s Multiple Comparison post hoc test (comparison between AS and NA group) or Kruskall-Wallis tests (comparison within the group) with Dunn’s Multiple Comparison post hoc test; * *p* < 0.05, ** *p* < 0.01,*** *p* < 0.001

In the next step, we investigated the effect of ALI-cultured mucociliary differentiated HBECs on the efficiency of the TGF-β_1_-induced myofibroblastic transitions of HBFs. Here, we analyzed the FMT potential of HBFs in compatible (AS/AS, NA/NA) and mixed (AS/NA, NA/AS) EMTU in comparison to the HBFs cultured alone (in control conditions or in the presence of the TGF-β_1_ (Fig. 3a). The exogenous TGF-β_1_ strongly increased the expression of the FMT-related genes such as *ACTA2* (Fig. 3b), *TAGLN* (Fig. 3c) and *FN1* (Fig. 3d) in the HBFs AS. Effects of the TGF-β_1_ on the expression of these genes in the HBFs NA were less evident, which is in accordance with our previous observations in standard ‘2D’ cultures [23, 26]. All the tested TGF-β_1_–induced FMT-related genes in the HBFs AS were much less expressed when cultured in the presence of mucociliary differentiated HBECs regardless their phenotype. Noticeably reduced the *ACTA2, TAGLN* and *FN1* expression was observed in the TGF-β_1_-treated HBFs AS when cultured in both the same-donor and mixed EMTU (Fig. 3b - d). Similarly, the presence of mucociliary differentiated HBECs in EMTU (AS/NA or NA/NA) caused that the expression of *ACTA2* and *TAGLN* slightly enhanced by the TGF-β_1_ was reduced in the HBF NA populations (Fig. 3b, c). However, the epithelial layer had no effect on the *FN1* expression in the TGF-β_1_-treated HBFs NA (Fig. 3d). All together these results suggest that the protective impact of differentiated HBECs on the TGF-β_1_-induced myofibroblastic transitions is more pronounced in the HBFs AS in comparison to the NA ones, probably due to the higher responsiveness of HBFs AS to TGF-β_1_ than HBFs NA under the mono-culture condition.

### HBFs prevent destabilization of the TGF-β_1_-treated mucociliary differentiated HBECs in the EMTU model

**Fig. 4:**
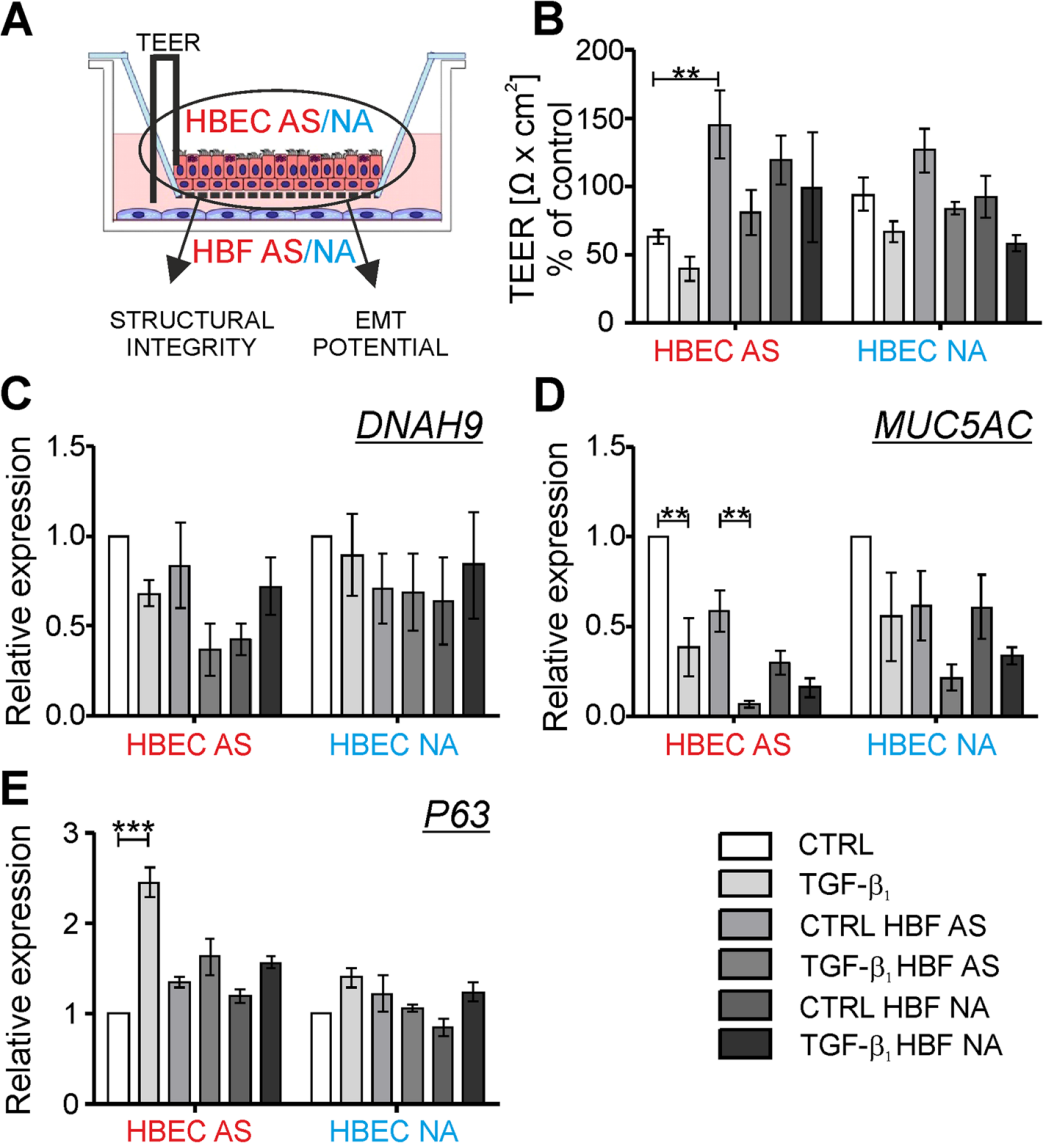
HBFs in EMTUs modulate the TEER value and ALI-related genes expression in AS epithelial layer. HBECs were cultured alone (CTRL, TGF-β_1_) or in **a** EMTU co-cultures in the absence (CTRL HBFs) or presence of TGF-β_1_ (5 ng/ml; TGF-β_1_ HBFs) for 4 days. TEER values were measured before EMTU establishment and after 4 days of cultures, in triplicates for each experimental condition. **b** Results were presented as the change of TEER value after 4 days of EMTU in relation to TEER values before EMTU establishment (% of control, where the control group was the differentiated HBECs cultured without the presence of HBFs AS or NA and TGF-β_1_) as an average ± SEM. **c-d** Expression of differentiated HBECs markers (*DNAH9*, *MUC5AC or P63*) in EMTU was determined by RT-qPCR (reference: *GAPDH* **c**-**d** or *GAPDH* and *18S* **e**). Results were presented as 2^-ΔΔCt^ value. Data from all experiments represent the mean ± SEM carried out on HBECs AS (*n* = 4) and NA (*n* = 4). Statistical significances were tested using the non-parametric Friedmann with Dunn’s Multiple Comparison post hoc test (comparison between AS and NA group) or Kruskall-Wallis tests (comparison within the group) with Dunn’s Multiple Comparison post hoc test; * *p* < 0.05, ** *p* < 0.01,*** *p* < 0.001

To check the effect of HBFs on the properties of differentiated HBECs in the EMTU (Fig. 4a), we compared tightness of the epithelial layer (Fig. 4b) and the expression of specific markers for the differentiated HBECs cultured alone and with the HBFs in the EMTU model (Fig. 4c-e). We observed diminished TEER values in the non-stimulated mucociliary differentiated HBECs AS in comparison to the NA counterparts. The response of ALI-differentiated HBEC populations to TGF-β_1_ administration was comparable in both tested groups (raw data presented in Fig. S1). However, tightness of the differentiated HBECs AS measured by TEER significantly increased in the presence of the HBFs from AS group. This effect was similar but less pronounced in the presence of the HBFs NA, but it was not observed in the differentiated HBECs NA in co-cultures with the HBFs derived from both study groups (Fig. 4b). Exposure of the HBECs AS to the TGF-β_1_ in the HBFs AS presence resulted in the reduction (c.a. 42%) of the TEER values, which indicated an unsealed structure of tight junctions in the AS epithelium (Fig. 4b). It was corresponding to the reduced expression of *DNAH9* and *MUC5AC* in this arrangement (Fig. 4c, d). The effect of the TGF-β_1_ on the epithelium tightness observed in the differentiated HBECs AS cultured in the presence of the HBFs AS was negligible, while in co-cultures of the differentiated HBECs AS with the HBFs NA it was noteworthy (Fig. 4b). It indicates a different effect of the HBFs AS or NA on the TEER values observed in the differentiated HBECs AS. The presence of the HBFs AS or NA had no significant effect on the *DNAH9* expression in both types of differentiated HBECs, but reduced the expression of *MUC5AC* in the differentiated HBECs AS cultured without the TGF-β_1_ (Fig. 4c, d). Expression of the basal cell marker – p63 transcription factor was significantly increased in the differentiated HBECs AS after the TGF-β_1_ treatment, but this effect was less pronounced in the HBECs NA. Moreover, the TGF-β_1_-induced enhanced expression of *P63* was attenuated in both tested group of the HBECs in the presence of the HBFs AS whilst the effect of HBFs NA was observed only in the TGF-β_1_-treated HBECs AS (Fig. 4e).

**Fig. 5:**
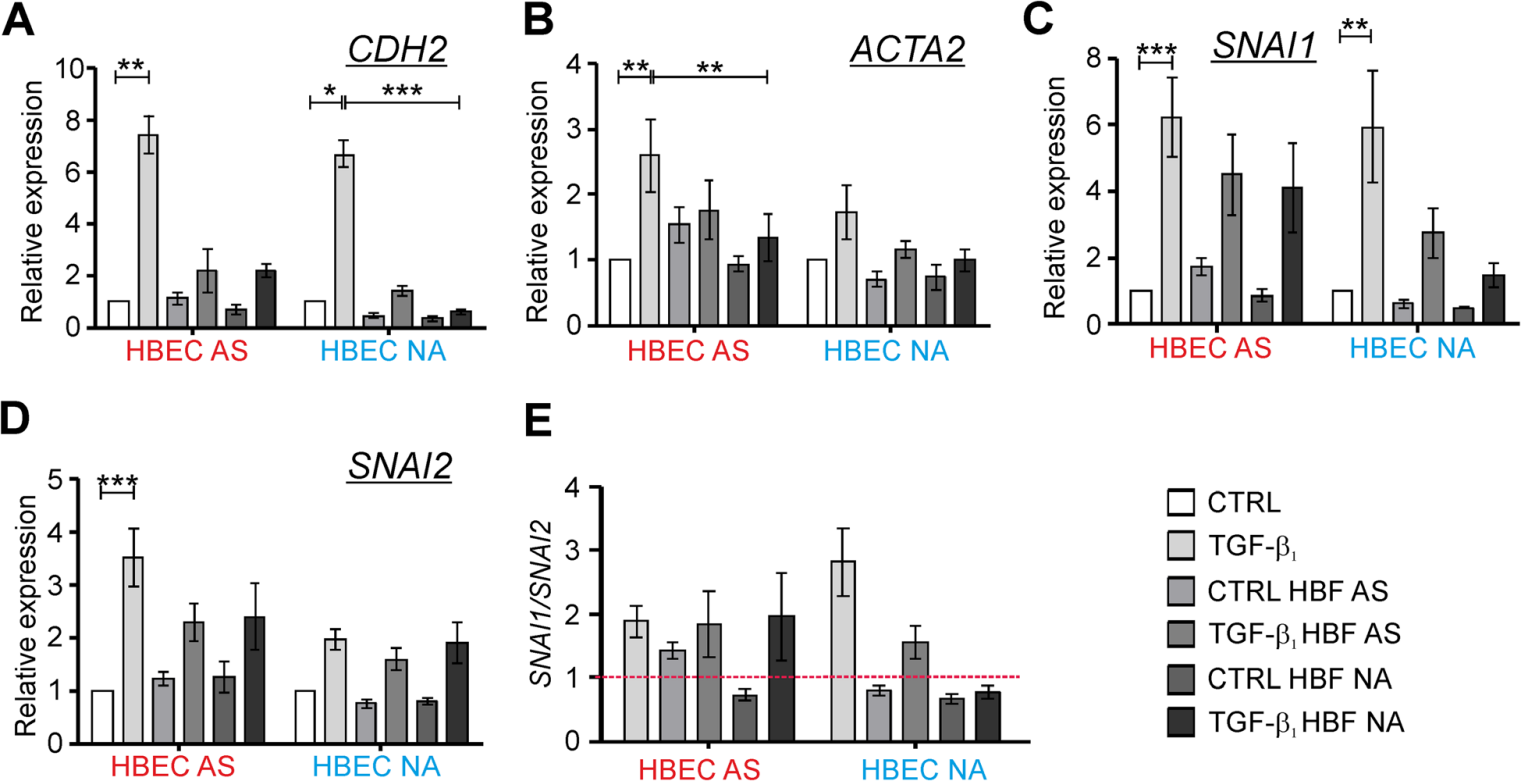
HBF populations in EMTU co-cultures protect the ALI-cultured HBECs against the potent TGF-β_1_ influence. HBECs were cultured alone or in EMTU in the absence (CTRL) or presence of TGF-β_1_ (5 ng/ml) for 4 days. Then, the expression of EMT-related genes: **a** *CDH2* (N-cadherin), **b** *ACTA2*, **c-d** *SNAI1* and *SNAI2* were determined by RT-qPCR using *GAPDH* as a reference gene and presented as 2^-ΔΔCt^ values. **e** *SNAI1/SNAI2* ratio was calculated and presented. Data from all experiments represent the mean ± SEM carried out on HBECs AS (*n* = 4) and NA (*n* = 4). Statistical significances were tested using the non-parametric Friedmann with Dunn’s Multiple Comparison post hoc test (comparison between AS and NA group) or Kruskall-Wallis with Dunn’s Multiple Comparison post hoc test (comparison within each group); * *p* < 0.05, ** *p* < 0.01,*** *p* < 0.001

Since the TGF-β_1_ is a well-known potent activator of cellular differentiation including EMT in epithelial cells, we investigated the expression of the EMT-related genes in the EMTU model. As shown in Fig. 5, mucociliary differentiated HBECs underwent the TGF-β_1_-induced EMT characterized by the significant enhanced expression of mesenchymal markers, as *CDH2*, *ACTA2*, *SNAI1* and *SNAI2*. The presence of the HBF populations from both tested groups affected the responsiveness of the HBECs AS to the TGF-β_1_ leading to noticeable reduction of the *CDH2* expression (Fig. 5a) in a comparable degree as in the HBECs NA. Similarly, the TGF-β_1_-induced *ACTA2* expression in the HBECs AS was significantly attenuated in the EMTUs with the HBFs NA and slightly weakened with the HBFs AS (Fig. 5b). The *ACTA2* expression was slightly expressed in the HBECs NA in response to the TGF-β_1._ Therefore, the effect of the HBFs presence was weaker. The TGF-β_1_-induced significant enhancement of the *SNAI1* expression was observed in both the differentiated HBECs AS or NA cultured alone. A noticeably diminished expression of *SNAI1* was observed only in the TGF-β_1_-treated differentiated HBEC NA co-cultures with HBFs from both study groups. However, the HBFs NA more effectively reduced the *SNAI1* expression than the AS ones (Fig. 5c). On the other hand, *SNAI2* was expressed in response to the TGF-β_1_ more efficiently in the HBECs AS than in the NA counterparts. Therefore, the presence of the HBF populations strongly affected the *SNAI2* expression only in the AS HBECs (Fig. 5d). We also compared the expression of *SNAI1* to *SNAI2* in our EMTU models. We showed that in the TGF-β_1_-treated differentiated AS HBECs cultured alone or in the presence of HBFs (AS or NA) the *SNAI1/SNAI2* ratio was comparable (Fig. 5e). However, the *SNAI1/SNAI2* ratio was meaningfully reduced in the populations of the TGF-β_1_-treated differentiated HBECs NA when cultured in the presence of HBFs from both study groups. Nevertheless, the HBFs NA more strongly reduced the ratio than in case of the AS ones (Fig. 5e). Our results indicate the protective effects of the HBFs on the structure of the TGF-β_1_–exposed mucociliary differentiated HBEC populations and their EMT potential.

## Discussion

In the light of clinical relevance, the in vitro EMTU model is much more accurate for conducting studies on the complex mechanisms of airway remodeling since it imitates in vivo-like conditions better than routinely using the monocultures of bronchial fibroblasts or bronchial epithelial cells cultured in vitro*.* The EMTU model was successively used in the studies on the interactions between epithelium and fibroblasts from healthy donors [35–37] or from donors with a diagnosed chronic lung diseases [38, 39] including asthma [16–18]. Nonetheless, until now the effect of the TGF-β_1_ on the structure and function of the asthmatic or non-asthmatic EMTU has been poorly described [35, 40]. Therefore, our study fills this gap since we have demonstrated for the first time that the asthmatic EMTUs (HBEC AS / HBF AS co-cultures) show a distinct response to the TGF-β_1_ treatment than the non-asthmatic ones and the effect of the TGF-β_1_ on the FMT efficiency of HBFs and the EMT potential in differentiated HBEC populations is weakened when these cells were cultured in the EMTU model. These results shed a new light on the Holgate’s EMTU concept indicating a greater role of intrinsic properties of HBFs in the regulation of the HBECs responsiveness to the TGF-β_1_ exposition as well as in modulation of the pseudostratified epithelium structure than previously thought [12, 41].

Comparison of the compatible asthmatic EMTU with the non-asthmatic ones presented in our study revealed more enhanced sensitivity to the TGF-β_1_ of the AS co-cultures than in case of the NA ones. Measurement of TEER determines the quality of the differentiated pseudostratified airway epithelium with well-developed tight junctions, and is often used as an epithelium barrier function marker [33, 34, 42, 43]. However, differences in the TEER values between ALI monocultures of asthmatic and non-asthmatic HBECs remain contradictory [44–47]. Our study revealed increased values of TEER in the non-stimulated ALI cultures of the HBECs NA in comparison to the AS counterparts. The HBFs presence in compatible EMTUs in the AS co-cultures strongly affects the TEER values in contrast to the NA compatible EMTUs. However, after the TGF-β_1_ stimulation of co-cultures, the AS EMTUs were characterized by a greater decrease of TEER than the NA ones, which indicated that the reaction of the AS differentiated HBECs to the TGF-β_1_ was intensified compared to the NA counterparts. Moreover, our study showed the enhanced TEER in the AS differentiated HBECs cultured in the presence of the HBFs regardless of their origin (AS or NA). The co-cultures of the HBFs with the HBECs NA slightly affected the TEER values. However, only the HBFs NA in the mixed TGF-β_1_ treated EMTU cultures showed a noticeable protective effect of the HBFs on the epithelial layer permeability (measured as the TEER value). A similar protective effect of fibroblasts on the TEER values was described previously in co-cultures of the HBECs with MRC5 cells in the EMTU model compared to equivalent HBEC monocultures [48].

Longitudinal studies on the TGF-β_1_-induced FMT potential (a key event in asthmatic subepithelial fibrosis) revealed a facilitated ability of the asthmatic HBF populations in standard ‘2D’ cultures for phenotypic shifts [23, 25, 27, 28, 30, 49], that may indicate the involvement of intrinsic properties of these cells in the regulation of FMT. In this study, we also observed the enhanced FMT potential in the TGF-β_1_-simulated HBF AS populations in contrast to the NA counterparts, when cells were grown in a monoculture as well as in compatible EMTUs. Moreover, a slightly larger decrease of the *ACTA2* expression was observed in the TGF-β_1_-treated HBFs AS after mixing EMTUs with the HBECs NA than in compatible counterparts. It indicates a protective effect of the non-asthmatic HBECs on the FMT potential in airway fibroblast populations which was previously reported by Reeves et al., [16, 17] in non-stimulated human lung fibroblasts. A substantial decrease of the *TAGLN* expression was observed in the TGF-β_1_-simulated HBFs AS cultured in the compatible and mixed EMTU and in the TGF-β_1_-simulated HBFs NA cultured in the compatible EMTU, but not in the EMTU with the HBECs AS. It suggests that the intrinsic properties of HBECs AS may stimulate the profibrotic response of HBFs NA. On the other hand, noticeable downregulation of the *FN1* expression analyzed in the TGF-β_1_-simulated AS HBFs after mixed and compatible EMTUs were carried out, was observed. However, a slightly increased expression of *FN1* was detected in the TGF-β_1_-simulated HBFs NA after mixed EMTU which may indicate that these interrelations between the tested cells affect the ECM protein production (*FN1*). A similar observation was described by Reeves et al., [18], where human lung fibroblasts from healthy donors expressed enhanced levels of *FN1* when cultured in the HBECs AS. Therefore, our results showed that the more effective FMT observed in the HBF AS populations can be considered as a universal and repetitive phenomenon in different types of ‘2D’ or 3D cultures and may be affected by the presence of both humoral stimuli such as the TGF-β_1,_ and interrelations of fibroblasts with the airway epithelium.

Differences between the asthmatic and non-asthmatic ALI-cultured pseudostratified HBECs have been also compared in these studies. We did not observe any significant differences at the mRNA level in the expression of *DNAH9* and *MUC5AC* between the differentiated HBECs AS and NA. However, we observed a slightly enhanced expression of *DNAH9* and notably increased expression of *MUC5AC* in the AS/AS EMTUs in comparison to the NA/NA EMTUs. Similar results that reported defective mucociliary clearance in asthmatics were observed previously [50–52]. The up-regulation of another marker of the basal cells subpopulation in the TGF-β_1_-treated pseudostratified epithelium – *P63* enhanced in the HBECs AS in comparison to NA, observed in our study, was diminished by the presence of the HBFs. The recent studies suggest that p63^+^ basal cells with stem cell-like properties are also involved in the induction and/or regulation of EMT significantly enhanced in asthmatic patients [31, 53] which remains in accordance with our observations.

Exposition of HBECs in monocultures or in AS EMTUs to the TGF-β_1_ insignificantly decreased the expression of the tested markers in subpopulations of differentiated HBECs leading to impairment of the barrier function of epithelium which complies with the previous reports [37, 40]. In the pseudostratified monocultures of the HBECs we observed the TGF-β_1_–induced EMT program described in lung fibrosis leading to the enhancement of the expression of mesenchymal genes as *ACTA2*, *CDH2* and EMT-related transcription factors *SNAI1* and *SNAIL2* [10, 31, 40, 54, 55]. A strong mesenchymal-like phenotype of the TGF-β_1_-stimulated HBECs AS was characterized by relatively high levels of mesenchymal markers (*CDH2*^*+*^and *ACTA2*^*+*^) and *SNAI1* and *SNAI2* expression (at a ratio ca. 2:1). However, when the pseudostratified TGF-β_1_-stimulated HBECs AS were co-cultured with HBFs, the expression of mesenchymal markers and *SNAI2* was noticeably reduced, but the *SNAI1/SNAI2* ratio remains comparable to the ratio observed in monocultures of the HBECs AS.

The recent reports have suggested that *SNAI1* preferably regulates α-SMA whereas *SNAI2* affects the epithelial-related genes [56]. Overexpression of *SNAI2* led to downregulation of E-cadherin and occludins in the HBECs, whereas overexpression of *SNAI1* to the enhanced α-SMA expression [40]. The elevated level of *SNAI1* leading to enhancement of α-SMA with the concomitantly enhanced expression of *SNAI2* which down-regulates the epithelial genes led to the efficient EMT of epithelial cells. However, a disturbed balance in the *SNAI1/SNAI2* ratio in conjunction with the function of *P63* may lead to incomplete EMT. In our study the TGF-β_1_-stimulated ALI-cultures of HBECs AS are characterized by the low *SNAI1/SNAI2* ratio with relatively high levels of the mesenchymal gene expression, whereas the HBECs NA showed an increase of this ratio and a relatively low expression of *ACTA2* with high *CDH2*. Johnson et al., [40] described a similar observation, where a relatively high ratio of *SNAI1/SNAI2* was observed in the HBECs from healthy donors (ca. 14), but in the HBECs isolated from mild and severe asthmatic patients it was significantly decreased (ca. 6.6 and 5.0 respectively) with a concomitant relatively high level of *CDH2* in all tested groups. Surprisingly, the *SNAI1/SNAI2* ratio in the TGF-β_1_-treated HBECs AS co-cultured with HBFs in the EMTU was comparable to the ratio in the TGF-β_1_-treated monocultures, but mesenchymal markers were significantly diminished in these cells. It suggests that not only the *SNAI1/SNAI2* ratio is involved in the EMT by modulation of the HBECs responsiveness to the TGF-β_1_, but also the HBFs intrinsic properties may have a protective effect on the HBECs reactivity. Despite that, our hypothesis requires further research to show which features of HBFs may have a significant effect on the EMT potential of the bronchial HBECs cultured in the EMTU.

## Conclusions

Functional interactions between HBECs and HBFs within the epithelial-mesenchymal trophic unit are necessary for a proper functioning of lung tissue. EMTU of HBECs and HBFs derived from AS patients and NA donors were established in this study. The effects of the TGF-β_1_ on the properties of HBECs cultured in the air-liquid-interface and effectiveness of FMT in HBFs in the EMTU models from both studied groups were compared and analyzed for the first time. Our results are the first to show that the AS co-cultures are more sensitive to the TGF-β_1_ than the NA ones. Our data also demonstrated a protective effect of HBFs on the properties of HBECs, which suggested that intrinsic properties of not only epithelium but also subepithelial fibroblasts affected a proper condition and function of the EMTU in both normal and asthmatic individuals. Our study suggests that EMTU AS and NA systems will be important tools for studying the mechanisms of subepithelial fibrosis in asthma, as well as for drug screening.

## Methods

### Patients characteristics

All patients were treated in the Faculty of Medicine of the Jagiellonian University Medical College, Krakow, Poland, and were in stable clinical conditions. The experimental group consisted of 4 patients with moderate asthma [Global Initiative for Asthma (GINA) grade 3–4; 4 females, age (years): 50.2 ± 18.7; BMI (kg/m^2^): 28.9 ± 4.0; FEV1 (% predicted): 107 ± 20.9; FEV1/FVC (%): 76.5 ± 7.15; iGCS (inhaled glucocorticoids; fluticasone) and long-acting beta agonists (LABAs); 4/4]. The control group of 4 non-asthmatic patients in whom diagnostic bronchoscopy ruled out any serious airway pathology [4 females, age (years): 40.5 ± 17.21; BMI (kg/m^2^): 26.6 ± 3.1; FEV1 (% predicted): 106.45 ± 9.42; FEV1/FVC (%): 81.11 ± 4.10]. The control subjects were referred for bronchoscopy due to diagnosis of cough, as a part of routine investigation. All controls were non-allergic, had normal lung function tests. Additionally, respiratory tract disorders (e.g. asthma, sarcoidosis, interstitial lung disease, COPD, lung cancer, etc.) were further ruled out during clinical investigation (e.g. no pathology in airway biopsy or CT scan). All study participants were non-smokers. The study was approved by the Jagiellonian University Ethics Committee (Decision No. 122.6120.16.2016; 22 January 2019) and informed written consent was obtained from all participants.

### HBFs cultures

Primary cultures of HBFs were isolated from bronchial biopsies derived from patients with diagnosed asthma (AS) and from non-asthmatic donors (NA) according to the protocols described previously [57]. HBFs were cultured in complete medium: Dulbecco’s Modified Eagle Medium (DMEM) with high glucose (4500 mg/L glucose; HG) supplemented by 10% fetal bovine serum (FBS, Gibco) and 1% penicillin/streptomycin (Sigma-Aldrich, St. Louis, MO, USA) in standard conditions (37 °C, humidified atmosphere, 5% CO_2_). Cells between 5^th^–10^th^ passages were used in the experiments. HBFs were plated at a density of 5000 cells/cm^2^ in complete medium for 24 h. Afterwards cells were incubated for the next 24 h in the serum-free medium: DMEM HG supplemented with 0.1% bovine serum albumin (BSA, Sigma-Aldrich) or in the medium dedicated for air-liquid interface (ALI) cultures (ALI medium; 1:1 mixture of Bronchial Epithelial Basal Medium (BEBM) and DMEM supplemented by BEGM™ SingleQuots™ Supplement Pack, Lonza). When indicated, the human recombinant TGF-β_1_ (5 ng/mL, stock prepared in 1 mg/mL BSA/PBS) was added.

### Mucociliary differentiation of HBECs

**Fig. 6:**
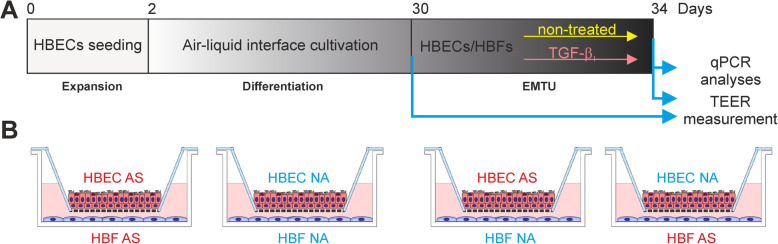
Schematic illustration of **a** the EMTU establishment and **b** all used cell combinations

After isolation from bronchial biopsies derived from the AS patients and NA donors described previously [29], HBECs were seeded onto collagen-coated flasks and cultured to ca. 90% confluence in Bronchial Epithelial Growth Medium (BEGM; BEBM with supplements). Then, cells were passaged and transferred onto collagen-coated Transwell polycarbonate inserts (0.4-mm pore size; Corning) at a density of 1.3–1.5 × 10^5^ cells/cm^2^ in BEGM. Next day, the medium from HBECs was removed and the medium below inserts was replaced by the ALI medium with all-trans-retinoic acid (50 nM; Sigma-Aldrich). The cells were cultured in the air-liquid interface (ALI) for 30 days (Fig. 6) to obtain a pseudostratified epithelium containing ciliary (*DNAH9*-positive) and goblet cells (*MUC5AC*-positive).

### Epithelial-mesenchymal trophic unit model

HBFs were seeded into 12-well plates in a complete medium. Next day the medium was replaced by the fresh ALI medium and inserts with the mucociliary differentiated HBECs were transferred into appropriate wells (in combinations as in Fig. 6b). The EMTU cultures were exposed to the TGF-β_1_ and cultivated by 4 days (Fig. 6a). A profibrotic response of HBECs and HBFs on the TGF-β_1_ was analyzed by RT-qPCR, immunofluorescence and/or *in-cell* ELISA assay.

### Transepithelial electrical resistance (TEER) measurements

A fresh medium was added to the ALI-cultured HBECs to the upper part of inserts and TEER was measured as described previously [33, 34] using Millicell ERS-2 Voltohmmeter (Merck Millipore) each in triplicates. Results were presented as a change in TEER values (Ω x cm^2^) in the mucociliary differentiated HBECs after 4 days of the EMTU as a  percentage of control, where the control was the same well before the EMTU establishment (Ω x cm^2^ after EMTU/Ω x cm^2^ before EMTU)*100%.

### Analyses of α-SMA intracellular localization and content

For immunofluorescent studies, the HBFs growing on coverslips were fixed with 3.7% formaldehyde/PBS, permeabilized with 0.1% Triton X-100/PBS and blocked with 3% BSA/PBS. Immunostaining of α-SMA was performed using primary anti-α-SMA antibody (mouse monoclonal IgG, A2547, clone 1A4, 1:400, Sigma-Aldrich) and compatible goat anti-mouse secondary IgG antibody conjugated with Alexa Fluor 488 (1:500, Thermo Fisher) with Hoechst 33258 (1 μg/mL, Sigma-Aldrich) for cell nuclei detection. Slides were mounted in the fluorescence mounting medium (DAKO) and visualized by automatic fluorescent microscope Leica DMI6000B (Leica Microsystems, Wetzlar, Germany), equipped with the LAS-X software, all at the same fluorescent time exposure. For the cell-based enzyme-linked immunosorbent (*in-cell* ELISA) analyses of the α-SMA and fibronectin (using a primary rabbit polyclonal antibody, F3648; 1:2000; Sigma-Aldrich) content, methanol-fixed HBFs were prepared according to the protocol described previously [57]. Results were presented as absorbance values measured at 450 nm by Microplate Reader (Thermo Fisher Scientific).

### Real-time quantitative PCR

After 4 days of the EMTU (as depicted in Fig. 1a-b), both types of cells were collected separately and the total mRNA was isolated according to the manufacturer’s protocol using the RNAx/miRNA GeneMATRIX UNIVERSAL purification kit (E3599–02, EURx). Afterwards, the reverse transcription reaction carried out using the NG dART RT-PCR Kit (E0801–02; EURx), 150 ng cDNA was used for further analyses. Expression of genes was performed using a real-time polymerase chain reaction (RT-qPCR, real-time PCR) with SYBR™ Green PCR Master Mix (Applied Biosystems, Thermo Fisher Scientific) and designed specific primers (Table 1). Results were presented as the 2^-ΔΔCt^ or 2^-ΔCt^ value ± SEM.

**Table 1:** Primers sequences

***GENE***	***SEQUENCE***			
*ACTA2*	F′	CTGTTCCAGCCATCCTTCAT	R’	CCGTGATCTCCTTCTGCATT
*DNAH9*	F′	GAGTCTTCCCAGCCACTCTTAC	R’	ATTCTGCATTCTCCAGAGCTTC
*GAPDH*	F′	GAAGGTGAAGGTCGGAGT	R’	GAAGATGGTGATGGGATTTC
*MUC5A*	F′	TTCCATGCCCGGGTACCTG	R’	CAGGCTCAGTGTCACGCTCTT
*CDH2*	F′	CTCCATGTGCCGGATAGC	R’	CGATTTCACCAGAAGCCTCTAC
*P63*	F′	CCCGTTTCGTCAGAACACAC	R’	CATAAGTCTCACGGCCCCTC
*SNAI1*	F′	GCTGCAGGACTCTAATCCAGA	R’	ATCTCCGGAGGTGGGATG
*SNAI2*	F′	TGGTTGCTTCAAGGACACAT	R’	GTTGCAGTGAGGGCAAGAA
*FN1*	F′	TGTGGTTGCCTTGCACGAT	R’	GCTTGTGGGTGTGACCTGAGT
*TAGLN*	F′	CGTGGAGATCCCAACTGGTT	R’	AAGGCCAATGACATGCTTTCC
*TNC*	F′	GGTCCACACCTGGGCATTT	R’	TTGCTGAATCAAACAACAAAACAGA
*18S*	F′	GTAACCCGTTGAACCCCAT	R’	CCATCCAATCGGTAGTAGCG

### Statistics

Data are presented as a mean ± SEM. Statistical significances the non-parametric Friedmann with Dunn’s Multiple Comparison post hoc test: * *p* < 0.05, ** *p* < 0.01, *** *p* < 0.001 (the comparison between the tested AS and NA group) or the Kruskall-Wallis with Dunn’s Multiple Comparison post hoc test (the comparison within the group): * p < 0.05, ** p < 0.01, *** *p* < 0.001. The statistical analyses were performed using GraphPad Prism 5.0 software.

## Supplementary Information

**Additional file 1: Figure S1.** A raw data from TEER measurement during the EMTU establishment and cultures. Tables contain a raw data collected during TEER measurement and converted according to appropriate guidelines in ALI-differentiated HBEC populations **(A)** before EMTU establishment (day 0), **(B)** after EMTU cultures (day 4) and **(C)** ratio of TEER measured in wells after/well before EMTU ((A/B)*100%) expressed in %.

## Data Availability

All data generated or analysed during this study are included in this published article.

## Abbreviations

α-SMA: α-smooth muscle actin
*ACTA2*: Smooth muscle α-2 actin gene
ALI: Air-liquid interface
ANOVA: Analysis of variance
AS: Asthmatic
BEBM: Bronchial epithelial basal medium
BEGM: Bronchial epithelial growth medium
BMI: Body mass index
BSA: Bovine serum albumin
*CDH1*: E-cadherin gene
*CDH2*: N-cadherin gene
DMEM: Dulbecco’s Modified Essential Medium
*DNAH9*: Dynein axonemal heavy chain 9 gene
ECM: Extracellular matrix
ELISA: Enzyme-linked immunosorbent assay
EMT: Epithelial-to-mesenchymal transitions
EMTU: Epithelial-mesenchymal trophic unit
FBS: Fetal bovine serum
FEV1: Forced expiratory volume
FMT: Fibroblast-to-myofibroblast transition
*FN1*: Fibronectin gene
FVC: Forced vital capacity
*GAPDH*: Glyceraldehyde 3-phosphate dehydrogenase gene
GINA: Global Initiative for Asthma
HBECs: Human bronchial epithelial cells
HBFs: Human bronchial fibroblasts
HG: High glucose
HLFs: Human lung fibroblasts
iGCS: Inhaled glicocorticosteroids
IgG: Immunoglobulin G
LABAs: Long-acting beta agonists
*MUC5AC*: Mucin 5 AC gene
NA: Non-asthmatic
PBS: Phosphate-buffered saline
RT-qPCR: Real-time quantitative polymerase chain reaction
SEM: Standard error of the mean
*SNAI1*: Snail transcription factor gene
*SNAI2*: Slug transcription factor gene
*TAGLN*: Transgelin gene
TEER: Transepithelial electrical resistance
TGF-β_1_: Transforming growth factor type β_1_
*TNC*: Tenascin C gene
