# Supplementary figures and images for: Responsiveness of human bronchial fibroblasts and epithelial cells from asthmatic and non-asthmatic donors to the transforming growth factor-β1 in epithelial-mesenchymal trophic unit model
Source: BMC Mol Cell Biol. 2021 Mar 12;22:19. doi: 10.1186/s12860-021-00356-8 (PMC7953709; doi:10.1186/s12860-021-00356-8)

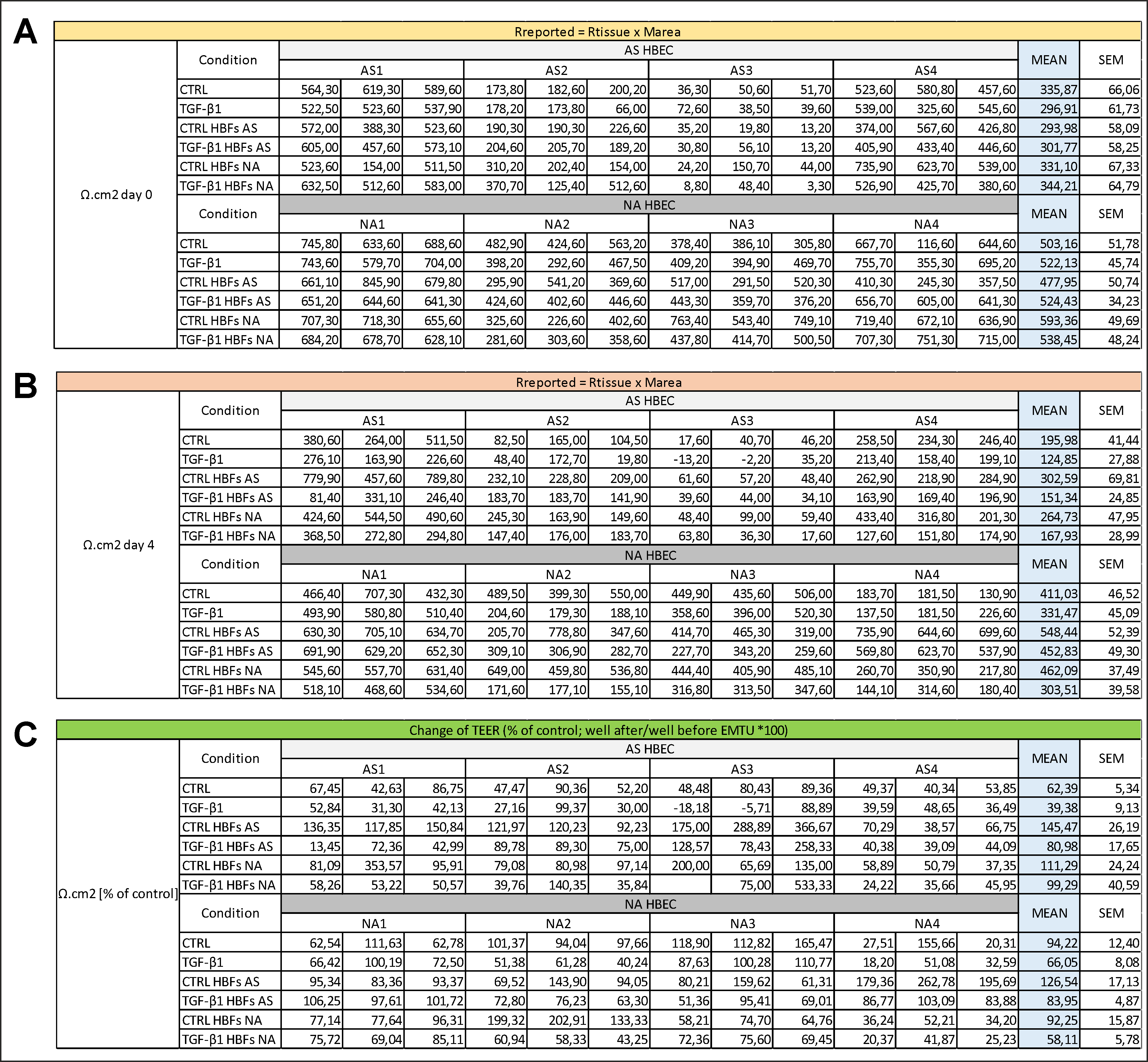

Supplement: Supplementary file 1 — Additional file 1: Figure S1. A raw data from TEER measurement during the EMTU establishment and cultures. Tables contain a raw data collected during TEER measurement and converted according to appropriate guidelines in ALI-differentiated HBEC populations (A) before EMTU establishment (day 0), (B) after EMTU cultures (day 4) and (C) ratio of TEER measured in wells after/well before EMTU ((A/B)*100%) expressed in %. [file 12860_2021_356_MOESM1_ESM.tif]
